# Supplementary material for: Genomic signatures suggesting adaptation to ocean acidification in a coral holobiont from volcanic CO2 seeps
Source: Commun Biol. 2023 Jul 22;6:769. doi: 10.1038/s42003-023-05103-7 (PMC10363134; doi:10.1038/s42003-023-05103-7)
Supplement: Supplementary file 2 — Supplementary Information [file 42003_2023_5103_MOESM2_ESM.pdf]

Supplementary Information

Synergistic genomic mechanisms of adaptation to ocean acidification in a coral holobiont

Carlos Leiva[1]*, Rocío Pérez-Portela[2,3], Sarah Lemer[1]

[1]University of Guam Marine Laboratory, 303 University Drive, 96923 Mangilao, Guam
[2]Departament de Biologia Evolutiva, Ecologia i Ciències Ambientals, Facultat de Biologia, Universitat de Barcelona, Av. Diagonal 643, 08028 Barcelona, Spain
[3]Institut de Recerca de la Biodiversitat (IRBio), Universitat de Barcelona, Barcelona, Spain

*Corresponding author: cleivama@gmail.com, ORCID: 0000-0003-4398-329X

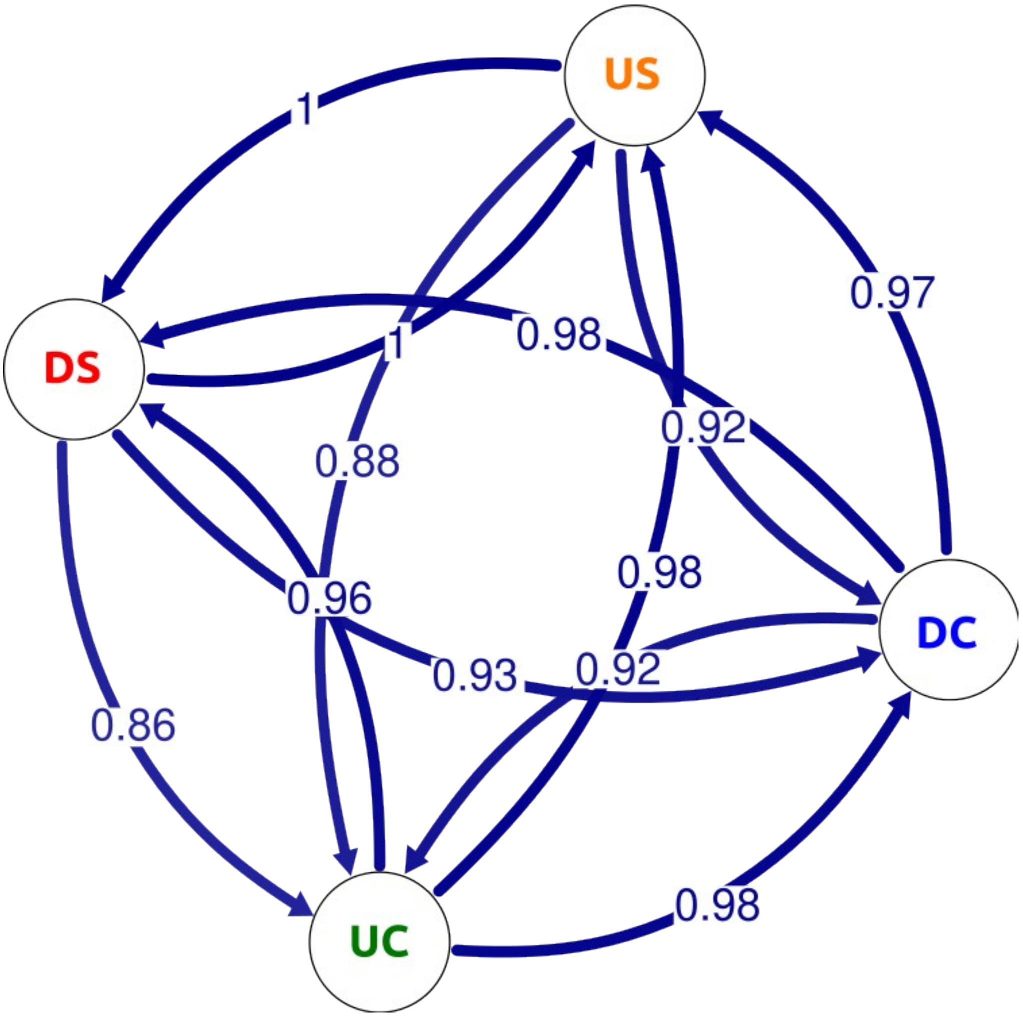

Supplementary Figure 1. *G*$_{ST}$ migration network. Circles represent sampling sites: red DS: Dobu Seep; blue DC, Dobu Control; orange US, Upa-Upasina Seep; green UC, Upa-Upasina Control.

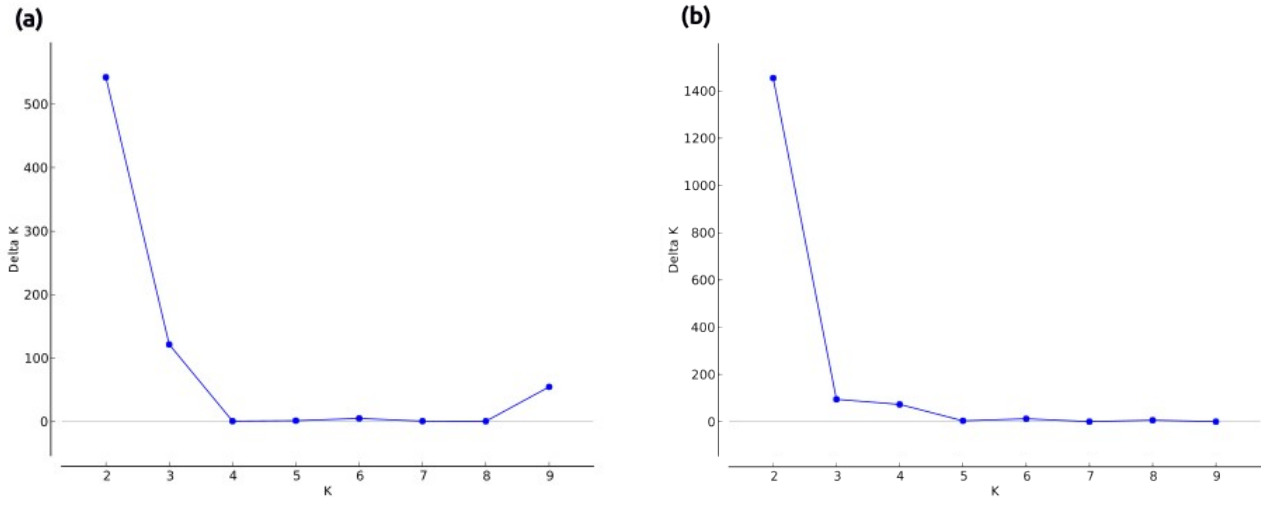

Supplementary Figure 2. Delta *K* plots for the STRUCTURE analyses. (a) Using the 11,169 independent neutral SNP dataset and (b) using the 625 candidate adaptive SNP dataset.

Revigo TreeMap Biological Process

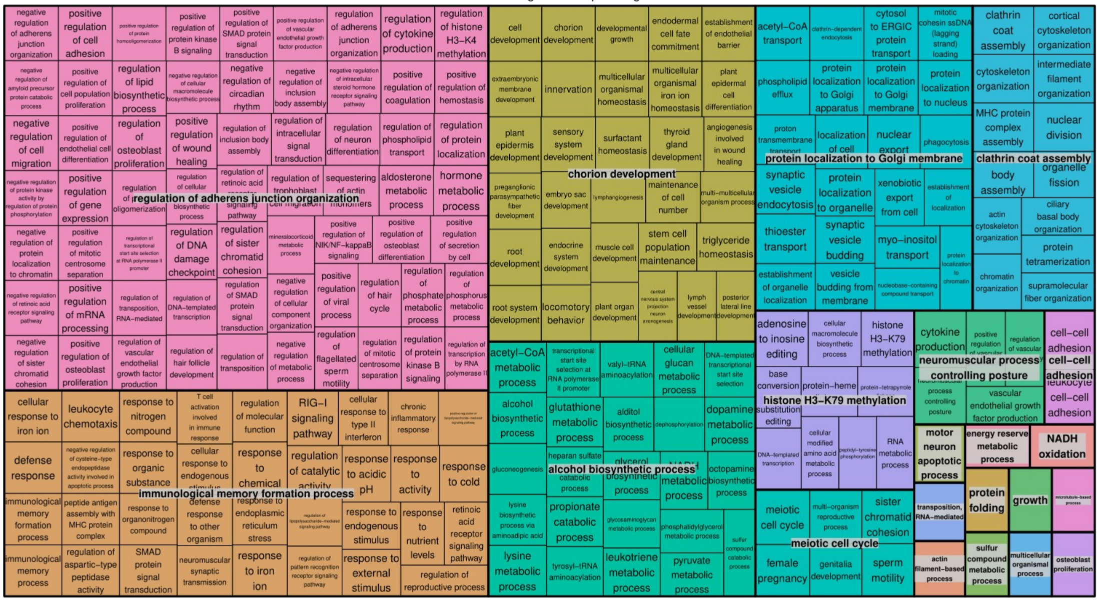

Revigo TreeMap Cellular Component

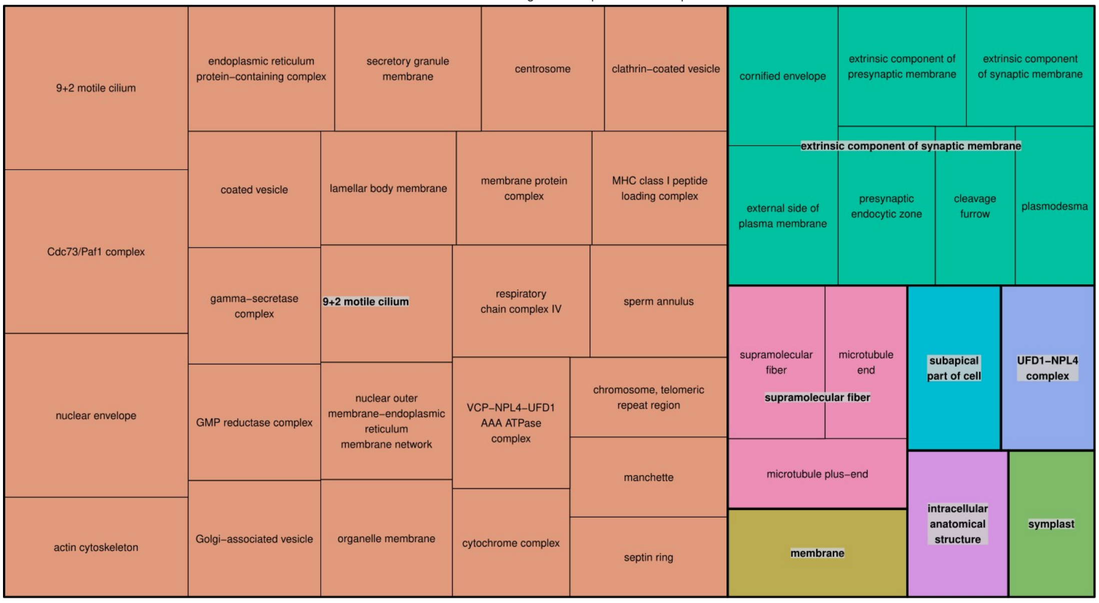

Revigo TreeMap Molecular Function

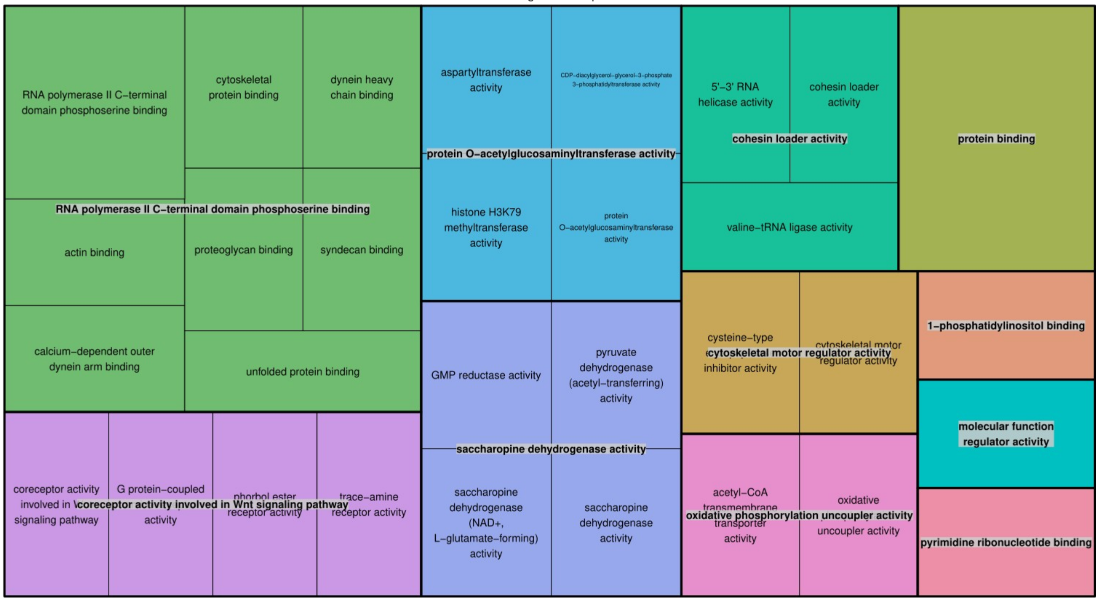

Supplementary Figure 3. REVIGO treemaps for Biological Process, Cellular Component and Molecular Function.
