# Supplementary material for: Genomic signatures suggesting adaptation to ocean acidification in a coral holobiont from volcanic CO2 seeps
Source: Commun Biol. 2023 Jul 22;6:769. doi: 10.1038/s42003-023-05103-7 (PMC10363134; doi:10.1038/s42003-023-05103-7)
Supplement: Supplementary file 3 — Description of Additional Supplementary Files [file 42003_2023_5103_MOESM3_ESM.pdf]

Description of Additional Supplementary Files

File name: Supplementary Data 1
Description: Complete list of candidate adaptive SNPs with their positions, names in the A. millepora reference genome, annotations, SnpEff results and E-value of the blast searches for the SNPs that fell in intergenic.

File name: Supplementary Data 2
Description: Gene Set Enrichment Analysis (GSEA) results.

File name: Supplementary Data 3
Description: Numerical source data for Figure 4.

File name: Supplementary Data 4
Description: Numerical source data for Figure 6b.
